# Drawing attention? Going through judgments regarding child and adolescent suicide attempts in emergency rooms from a professional perspective

**DOI:** 10.1590/1980-220X-REEUSP-2023-0281en

**Published:** 2024-02-12

**Authors:** Danton Matheus de Souza, Danila Maria Batista Guedes, Gabriella de Andrade Boska, Niflyer Costa Miranda, Lisabelle Mariano Rossato

**Affiliations:** 1 Universidade de São Paulo, Escola de Enfermagem, Programa de Pós-Graduação em Enfermagem, São Paulo, SP, Brazil.; 2 Universidade Federal do Rio Grande do Sul, Escola de Enfermagem, Departamento de Assistência e Orientação Profissional, Rio Grande do Sul, RS, Brazil.; 3 Universidade de São Paulo, Escola de Enfermagem, São Paulo, SP, Brazil.; 4 Universidade de São Paulo, Escola de Enfermagem, Departamento de Enfermagem Materno Infantil e Psiquiátrica, São Paulo, SP, Brazil.

**Keywords:** Suicide, Attempt, Emergencies, Child Health, Child, Adolescent, Health Personnel, Intento de Suicidio, Urgencias Médicas, Salud Infantil, Niño, Adolescente, Personal de Salud, Tentativa de Suicídio, Emergências, Saúde da Criança, Criança, Adolescente, Pessoal de Saúde

## Abstract

**Objective:**

To understand the perceptions of the multidisciplinary team of an emergency department regarding the care of children and adolescents who have attempted suicide.

**Method:**

An exploratory-descriptive, qualitative study, in light of the Symbolic Interactionism theoretical framework. Thirteen professionals from the multidisciplinary team from two emergency rooms (children and adults) of a secondary hospital in São Paulo participated. Data were collected between August and September 2018 using semi-structured interviews, analyzed using thematic content analysis complemented by the IRAMUTEQ^®^ software.

**Results:**

Two central categories emerged: Multidisciplinary team perceptions regarding attempted suicide care; and Multidisciplinary team perceptions regarding the possibilities for improving attempted suicide care. From these, professional perceptions of care, risk factors, emotional reactions, limitations of emergency rooms and strategies for improving practice were observed.

**Conclusion:**

Professionals perceived suicide attempt care from a biomedical and reductionist perspective, with an approach marked by stigma, judgment and lack of preparation.

## RESUMO

**Objetivo:**

Compreender as percepções da equipe multiprofissional de um departamento de urgência e emergência diante do cuidado à criança e ao adolescente por tentativa de suicídio.

**Método:**

Estudo exploratório-descritivo, qualitativo, à luz do referencial teórico do Interacionismo Simbólico. Participaram 13 profissionais da equipe multiprofissional de dois prontos-socorros (infantil e adulto) de um hospital secundário de São Paulo. Os dados foram coletados entre agosto e setembro de 2018 utilizando entrevista semiestruturada, analisados pela análise temática de conteúdo com complementação do *software* IRAMUTEQ^®^.

**Resultados:**

Emergiram duas categorias centrais: Percepções da equipe multiprofissional diante do cuidado à tentativa de suicídio; e Percepções da equipe multiprofissional diante das possibilidades para o aperfeiçoamento do cuidado à tentativa de suicídio. A partir dessas, observaram-se as percepções dos profissionais diante do cuidado, fatores de risco, reações emocionais, limitações dos prontos-socorros e estratégias para aperfeiçoamento da prática.

**Conclusão:**

Os profissionais perceberam o cuidado às tentativas de suicídio sob uma ótica biomédica e reducionista, com abordagem marcada por estigma, julgamento e falta de preparo.

## RESUMEN

**Objetivo:**

Comprender las percepciones del equipo multidisciplinario de un servicio de urgencia y emergencia sobre la atención de niños y adolescentes por intento de suicidio.

**Método:**

Estudio exploratorio-descriptivo, cualitativo, a la luz del marco teórico del Interaccionismo Simbólico. Participaron trece profesionales del equipo multidisciplinario de dos salas de emergencia (infantil y de adultos) de un hospital secundario de São Paulo. Los datos fueron recolectados entre agosto y septiembre de 2018 mediante entrevistas semiestructuradas, analizados mediante análisis de contenido temático complementado con el software IRAMUTEQ^®^.

**Resultados:**

Emergieron dos categorías centrales: Percepciones del equipo multidisciplinario sobre la atención al intento de suicidio; y Percepciones del equipo multidisciplinario sobre las posibilidades de mejorar la atención al intento de suicidio. A partir de ellos, se observaron percepciones de los profesionales sobre la atención, factores de riesgo, reacciones emocionales, limitaciones de las salas de emergencia y estrategias para mejorar la práctica.

**Conclusión:**

Los profesionales percibieron la atención al intento de suicidio desde una perspectiva biomédica y reduccionista, con un enfoque marcado por el estigma, el juicio y la falta de preparación.

## INTRODUCTION

Children’s mental health represents a challenge to public health. There are worldwide estimates that more than 20% of children and adolescents experience some mental health problem, with an approximate rate of more than a thousand visits to specialized services for every 100 thousand children and adolescents^(1)^. This number may still be underestimated, considering that, frequently, this public only enters the Psychosocial Care Network (RAPS - *Rede de Atenção Psicossocial*) after experiencing a crisis or psychiatric emergency situations, such as suicide attempts (SA)^(2)^.

SA can be defined as any non-fatal suicidal behavior, with the intention of ending one’s life, being a multifactorial phenomenon resulting from an interaction of biological, psychological, cultural and environmental factors^(2,3)^. The World Health Organization (WHO) indicates that, annually, more than 700,000 people commit suicide, and estimates that for each death there are more than 20 SA (estimated to be more than 14 million SA). Furthermore, suicide is the third and second cause of death among adolescents aged 10 and 19 and 15 and 19, respectively^(4)^.

In Brazil, in a cross-sectional study, carried out with the hospital information system with children between five and nine years old, it demonstrated that, from 2006 to 2017, there were 1,994 hospital admissions due to SA^(5)^. In adolescents, a similar investigation indicated that, between 2007 and 2016, 12,060 young people between ten and 19 years old were admitted for SA in Brazil^(6)^. Another study carried out in an emergency department over five years identified 88 adolescents who were treated for SA^(2)^. Although self-inflicted violence is compulsory to report, in Brazil, there is still underreporting or use of codes related to the means used for the attempt, such as poisoning or trauma (accidents), which contributes to an unreliable estimate^(2)^. Part of this aspect occurs due to the difficulty in social recognition that children and young people may intend to take their own lives^(7)^.

The social representation of children, seen as angelic, kind and happy figures, may be one of the reasons why SA is not identified, being “incompatible” with this stage of life^(2,5)^. Adolescents, despite experiencing a transition stage, with psychological changes (construction of identity, originality and self-image) and social changes (search for autonomy, acceptance and occupation in collective spaces), which can lead to impulsive behaviors, are still perceived as “problematic” through the “teenage boredom” phase^(8)^.

Recognizing the importance of the topic, Brazil has moved forward with public policies, but it is still necessary to invest in the implementation of guidelines provided for in policies both in the context of RAPS and in dialogue with other sectors. In the scope of mental health, there is the Child and Youth Mental Health Policy (PSMIJ - *Política de Saúde Mental Infantojuvenil*), based on individual care guidelines, universal welcoming, implicated and responsible referral, permanent construction of the network with intersectorality, territorialization and shared construction of mental health needs^(9)^. Specifically in SA, there is the Brazilian National Self-Mutilation and Suicide Policy, which aims to promote mental health, prevention and prevention of the phenomena as well as notification and coordination in the intersectoral network for comprehensive care^(10)^. However, policy formulation does not guarantee qualified care; a collective effort is required, with scientific research being vital for translating policies into clinical practice.

Although primary care and the Child and Youth Psychosocial Care Centers (CAPSIJ - *Centros de Atenção Psicossocial Infantojuvenil*) are the network’s organizers, as a gateway into RAPS for mental health care for children and adolescents^(9)^, approximately one third of this population is initially treated in emergency rooms (ER) by SA. This aspect is already recognized in policies, with emergency services being RAPS devices^(11)^, making it necessary to explore SA care in these spaces, essentially health professional perceptions, who are essential figures in providing dignified and quality care that strengthens the care network.

Scientific literature has previously explored SA perceptions among primary and secondary care health professionals^(12,13)^. It is imperative to continue advancing the literature, with this study focusing on professionals working in ER. In a context where global health services aim to reduce SA, aiming to achieve quality health and well-being of the population (third objective of sustainable development)^(14)^, and Brazil is restructuring after a setback in mental health policies, visibility of child and adolescent SA is emerging. Recognizing professional perceptions allows us to guide strategies for giving new meaning to the context and moving forward to guarantee these aspects. Thus, the following research question emerged: what are the health professional perceptions regarding SA care for children and adolescents in the ER? This study aimed to understand the perceptions of the multidisciplinary team of an emergency department regarding the care of children and adolescents due to SA.

## METHOD

### Study Design

This is an exploratory-descriptive study with a qualitative approach, based on the COnsolidated criteria for REporting Qualitative research guidelines^(15)^.

### Study Place and Population

Data were collected between August and September 2018 in an emergency department of a public teaching hospital in the city of São Paulo. The department is made up of two ER, one for children (newborns and adolescents under 15 years old) and one for adults (15 years old and under 19 years old). Participants were professionals from the multidisciplinary team (nursing team; doctors and social workers) working in the sectors and providing support in cases of SA.

### Selection Criteria

Professionals from the multidisciplinary team who had already experienced direct care for children or adolescents through SA in the emergency department (doctors, nurses, nursing technicians and social workers) were included. Eligible professionals were asked whether they had already treated at least one case of child and adolescent SA in the ER. Students, interns and residents were excluded, considering that they are not part of the sector’s permanent team, in addition to sector heads, as they are away from care, and professionals who were on vacation or leave during the collection period.

### Data Collection

Sampling was conducted using the convenience technique, on days when the researcher was available to be present at the service, with an individual approach to each health professional, presenting the objective of the study and inviting those who met the eligibility criteria. If accepted, a meeting was scheduled for data collection, in person. On the scheduled day, participants’ boss was notified that they would not be in the department to conduct the interview. After release, professionals were taken to a private location, provided by the co-participating institution.

Initially, a questionnaire was applied with professional characterization variables (sex, age, time since training and additional training). Afterwards, a semi-structured interview began, conducted by the following guiding question: what is it like for you to care for a child or adolescent with SA? By exploring the discourse according to the participants’ responses, it was possible to approach care, risk factors, feelings generated, limitations and perceptions regarding changes. The guiding question emerged through a consensus among researchers based on the objective of the study. The interviews were carried out by a female researcher, doctoral candidate in health sciences, with previous experience in conducting qualitative research. It is noteworthy that the researcher had no previous connection with participants. The audios were recorded by an electronic device, generating a total of seven hours, with an interview time between 17 and 45 minutes, and transcribed in full by the researcher.

There were no refusals to participate, no repeated interviews, and transcripts were not sent to participants. Data collection was completed when the objective of the study was achieved, without new additions of themes, according to the theoretical data saturation technique^(16)^. It is noteworthy that saturation was previously agreed upon by the study researchers, through a consensus.

### Methodological Framework

To understand professional perception, we chose to analyze the data in light of Symbolic Interactionism^(17)^, which allows exploring the phenomenon based on its symbolic construction, through the interactions that professionals experience in a social dynamic in which they are immersed, influencing their actions, definitions and beliefs. In this immersion, the professional is seen as an agent participating in their own experiences. Through social interactions (emergency context, child or adolescent cared for by SA, family members and companions, other professionals), human action (SA care), the self (construction of beliefs, opinions and perceptions) and the mind (interpretation of the phenomenon), symbols are constructed (perceptions about SA).

### Data Analysis and Treatment

The data were analyzed using Bardin’s thematic content analysis^(18)^. Data were skimmed (five to 10 times), codes and units of meaning were extracted and subsequently grouped into theoretical categories and subcategories. It is noteworthy that this first analysis was carried out in pairs, independently, with subsequent discussion and deliberation of categorization together with the other researchers, reaching a consensus. In order to complement the analysis, the data was processed using the *Interface de R pourles Analyze Multidimensionnielles de Textes et de Questionnaires* (IRAMUTEQ^®^) software, with a lexical analysis, allowing a quantitative look at qualitative data. To this end, the data were organized into textual *corpora*, identifying text segments with grouping of words with statistical difference (p < 0.05). For presentation, we chose the Descending Hierarchical Classification (DHC), using the chi-square test (x2), with the organization of words into classes, and similarity analysis (SA), with the visual representation of the connections between the words^(19)^. It is noteworthy that thematic analysis and lexical analysis, from an interactionist perspective, were integrated, allowing for in-depth exploration. Thus, the theoretical categories (thematic content analysis) and classes (IRAMUTEQ^®^) were named similarly.

### Ethical Aspects

The study was approved by the Research Ethics Committee of the School of Nursing at the *Universidade de São Paulo* (EE-USP), under Process 2,712,870 of June 14, 2018, and by the co-participating institution, under Process 2,778,446 of July 20, 2018. The professionals who agreed to participate signed the Informed Consent Form (ICF) in two copies, and were identified by the initial letters of the profession, such as SW (social worker), N (nurse), NT (nursing technician) and D (doctor), in addition to the respective number in ascending order of inclusion.

## RESULTS

**Figure 1:**
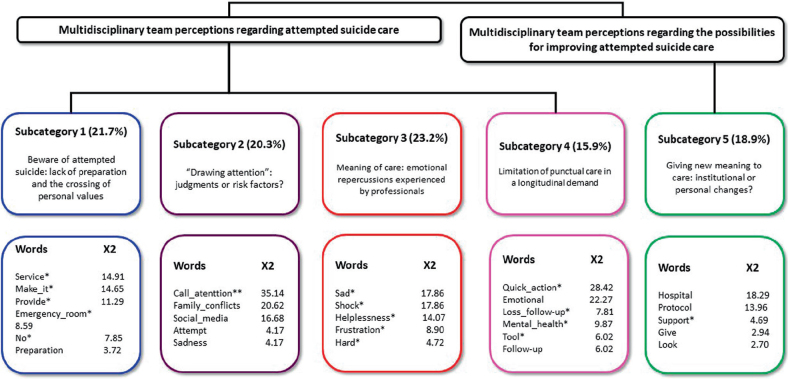
Descending Hierarchical Classification dendrogram with interconnection of the main categories and their subcategories – São Paulo, SP, Brazil, 2018. Note: X^2^ – Chi-square test; **p < 0.001; *p < 0.05.

A total of 13 professionals participated, including five nurses, three nursing technicians, two social workers and three doctors. Eleven were female; age ranged from 26 to 61 years, with an average of 36 years; The length of time in the profession and immersion in the study site ranged from five to 30 years, with an average of 15 years. Nine had *lato sensu* training, and five, *stricto sensu*. Lexical analysis generated 95 text segments, with 72.6% utilization, with 2,721 occurrences (words, forms or vocabulary), interconnecting the two theoretical categories generated by thematic content analysis, with interconnected subcategories (Figure 1).

### Category: Multidisciplinary Team Perceptions Regarding Attempted Suicide Care

Professionals realize that SA care (p < 0.05) for children and adolescents has increased in recent years and that the team conducts it appropriately. They mentioned that care is reflected by focusing on the physical with the aim of saving life. However, the word “no” (p < 0.05) was associated with a lack of knowledge and preparation for providing support for mental distress. The recognition of this limitation reflected in the approach of distancing children and adolescents, or an approach permeated by stereotypes: “*It’s common for people to say things like: “you’re a very beautiful, very good person, you’re studying, why do that?!” (SW5)*”. When justifying their conduct, professionals perceive the ER (p < 0.05) as a place to promote life, and, when caring for SA, personal values stand out. Furthermore, professionals mentioned that welcoming depends on a personal characteristic and not an action that permeates the team (Subcategory 1: Beware of attempted suicide: lack of preparation and the crossing of personal values) (Chart 1).

**Chart 1:** Professional speeches referring to subcategories 1 and 2 – São Paulo, SP, Brazil, 2018.

Category: Multidisciplinary team perceptions regarding attempted suicide care
**Subcategory 1: Beware of attempted suicide: lack of preparation and the crossing of personal values**
*Here it is perfect. It has a humanized team.* (...) *doctors save a lot.* (NT10) *In the emotional part, we have no tools. Everyone acts the way they think is best.* (...) *there are professionals who treat SA as a problem, but others say that it is a lack of shame, of being beaten, of pulling the ear.* (N2) *It is hard. It’s not the best service you like to give. We like to serve people who are really suffering from an illness, something that you seek to remedy. For someone who attempts suicide, you* (professional) *will only take action to get them out of the chaos they find themselves in. But it’s not a satisfaction, you say, “Damn, you came here brought by someone who is distressed”. It is a lack of responsibility by adolescents. A total alienation of the implications.* (...) *you provide care out of obligation, because it is your job.* (D11) *I always talk to myself: both an old man with an incurable disease fighting to live and a child, who has his whole life ahead of him, wanting to take his own life.* (N2) *I observe and approach the children. I see it as something integral to my role. Accept this type of complaint. It is an emotional pain that deserves respect and adequate care.* (N13)
**Subcategory 2: “Drawing attention”: judgments or risk factors?**
*I don’t have that knowledge of basic psychology to be able to differentiate whether the child has an ideation or is attracting attention.* (D7) *We went to talk to the mother. She said it wasn’t the first time, that the adolescent always did this to get attention.* (NT10) *The triggering factors alleged by the child are banal.* (D7) *It cannot be classified as SA; in fact, it is closer to ‘attempting to attract attention’. They (adolescents) warn, say what they did, and those who want to commit suicide don’t do that.* (D11) *I think there is a lack of attention from the family.* (N2)

In subcategory 2: “Drawing attention”: judgments or risk factors? (Chart 1), professionals indicated their perceptions about the triggering factors of SA in children and adolescents. It is noted that the recognition of emotional pain did not result in health care, but rather in more judgment, with “drawing attention” (p < 0.001) associated with the cause of SA. Furthermore, they indicated that SA occurs due to the lack of emotional control, the child or adolescent wanting to behave like an adult and having an immediacy in the search for fulfillment in life and a low threshold for frustration, which leads to a minimization of mental distress associated with SA. The trial follows the family, who are found guilty of not seeking services in advance or not retaliating against their child.

Professionals defined SA care as challenging, as it has repercussions on personal emotional reactions, such as frustration (p < 0.05), helplessness (p < 0.05), anguish, sadness (p < 0.05), shock (p < 0.05), revolt and doubts. Only one professional indicated satisfaction with care linked to clinical success, and another who indicated little emotional repercussions due to previous experiences in care (Subcategory 3: Meaning of care: emotional repercussions experienced by professionals) (Chart 2).

**Chart 2:** Professional speeches referring to subcategories 3 and 4 – São Paulo, SP, Brazil, 2018.

Category: Multidisciplinary team perceptions regarding attempted suicide care
**Subcategory 3: Meaning of care: emotional repercussions experienced by professionals**
*I confess that I didn’t understand why people would go to a hospital if they wanted to die, because here we are working to save lives, and those who want to take their lives come. This made me uncomfortable, because I was busy with other demands.* (N13) *At first, it was really annoying, I was angry.* (...) *I would enter the emergency room irritated, “What the hell, life is so good and this person is going to destroy life”.* (N6) *It’s frustrating. Service means that several things failed to get there.* (D7) *It makes quite an impact.* (...) *it doesn’t cause me suffering, because I’m already prepared, I’ve handled situations like this for many years, giving me structures to deal with.* (SW4)
**Subcategory 4: Limitation of punctual care in a longitudinal demand**
*We do what an emergency room can do.* (...) *we will carry out the procedures and then we will provide welcoming if the scenario allows.* (N13) *The adolescent has a first visit to the emergency room, and that’s it. Theoretically, this is what he needs here* (clinical care). (NT8) *Network interconnection is important. They are treated in the emergency room, but it is punctual.* (SW4)

In subcategory 4: Limitation of punctual care in a longitudinal demand (Chart 2), only one professional indicated the ER as a potential place to act on mental distress demands, while another 12 perceive that “quick action” (p < 0.05) and occasional psychiatric emergency limited care. When they performed welcoming, they reported that it occurred quickly, due to the lack of preparation, privacy, space, presence of family or understanding that only professionals specialized in mental health (p < 0.05) could carry out this approach. They requested interconsultations with psychiatrists, demanding that the child or adolescent be transferred to another service that is often specific to adult and elder care, and, upon returning, they observed that the main actions were referral to RAPS and medicalization. After care, professionals noticed a loss of follow-up (p < 0.05), indicating that there is no return on what was done in other services, with a lack of coordination between care networks.

### Category: Multidisciplinary Team Perceptions Regarding the Possibilities for Improving Attempted Suicide Care

**Figure 2:**
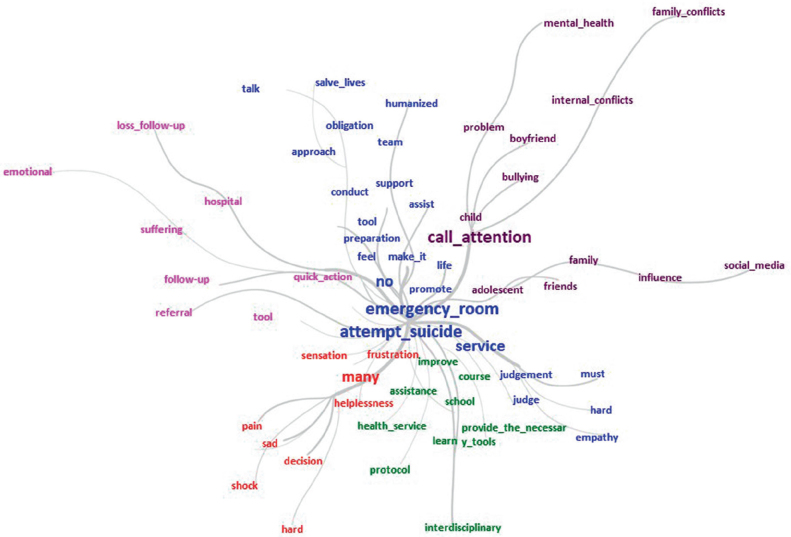
Similarity tree – São Paulo, SP, Brazil, 2018.

Professionals perceive the construction of an institutional protocol (p < 0.05), active search for children and adolescents with risk factors, support (p < 0.05) from mental health professionals, continuing education, with classes, courses, simulations, psychological support for the team and creation of a single team to provide care as strategies for improving care. Furthermore, professionals reflected that, as accepting mental distress is a personal attitude, such measures could have no effect in practice (Subcategory 5: Giving new meaning to care: institutional or personal changes?) (Chart 3). Figure 2 shows the similarity tree, which demonstrates the connections of words.

**Chart 3:** Professional speeches referring to subcategory 5 – São Paulo, SP, Brazil, 2018.

Category: Multidisciplinary team perceptions regarding the possibilities for improving attempted suicide care
**Subcategory 5: Giving new meaning to care: institutional or personal changes?**
*I think something had to be done to assess the extent to which this person actually has suicidal ideation or is drawing attention in a wrong way.* (D7) *Here we work very much based on protocols.* (NT9) *A protocol with interdisciplinary involvement is needed.* (...) *so that you can be a little more certain that, when you leave, there will be no new attempts.* (N3) *We have a lack of preparation in many areas. I may be unprepared to make a bandage, respond to a stop, but I train and I can improve. What I notice in practice is that people don’t care about learning what they can do. So, it depends more on her to raise awareness empirically and take the precautions that everyone should.* (N13)

## DISCUSSION

The professionals in this study realized that initial SA care is marked by limitations in welcoming and longitudinal care. It is reflected that this perception of the phenomenon is the result of social interactions^(17)^, where these professionals may have been influenced by the stigmatizing view of mental health, with judgments and moralism towards children and adolescents, with demands beyond the physical nature, cared for in an ER. This perception can be attributed to organizational and institutional difficulties and lack of knowledge about the phenomenon.

In fact, the ER service is not a promising place for long-term actions, mainly because mental health interventions require attention, active listening and bonding, challenges for emergency professionals due to the biomedical focus, complications and patient turnover. However, welcoming can and should begin in this location, already recognized as a RAPS device. Thus, the question arises: why are ER not seen as powerful places to provide mental health support for children and adolescents in SA care?

The PSMIJ recognizes, in its principle of universal access, that welcoming, listening and recognizing the legitimacy of a search for care is a form of care^(9)^, corroborating the WHO, which reiterates that welcoming SA must be initiated in the ER^(4)^, this service being recognized as a RAPS device^(11)^. The first welcoming is a difference, as it allows children and adolescents to recognize the health service as a source of support for mental distress^(2,12)^.

The absence of welcoming reflects on adherence to interventions, with rates of loss to follow-up of children and adolescents with SA between 25 and 80%^(20)^. On the other hand, those who follow up at a CAPIJ report that, initially, numerous emotional reactions made them move away from the service, however, over time and with welcoming interventions, the service was seen as a place that enhances changes, with adherence^(21)^.

There is a need to reflect on ways of welcoming people into an ER. A methodological investigation, with guideline construction and validation for multidisciplinary care for child and adolescent SA in the ER, indicated that welcoming must be carried out promptly and with quality, in a private room, with active listening, without the need for advice or speech, with an attitude empathetic, without minimizing emotional pain, started from the first clinical anamnesis after stabilization of the physical and mental clinical picture, identifying risk and protective factors. This approach can be carried out by different team members, without the need for a specialist professional as a mediator, as it can initiate a brief therapeutic intervention, with relapse prevention guidelines and looking at the dyad^(3)^.

In theory, the aforementioned guidelines may seem simple and easy to articulate in clinical practice, however, without actions for their effective translation, SA care will continue to be marked by a lack of preparation, limiting interventions and judgment. In this study, it is noted that professionals do not recognize themselves as instruments of care, devaluing soft technologies instead of hard technologies, expressing the need for instruments and protocols to guide clinical conduct in SA.

As Symbolic Interactionism predicts, individuals’ actions will occur according to the meaning that the phenomenon has for them^(17)^; in this case, professionals see SA as a burden, an obligation, and not a real demand for emerging health care, thus, they do not expend efforts on therapeutic interventions. Furthermore, it indicates that different individuals can define the same situation in different ways^(17)^, as seen in Chart 1, when comparing speeches from D11 and N2 with N13, who demonstrates an empathetic view of adolescents’ mental distress, showing himself available for initial welcoming. In this study, the majority of professionals show judgment towards SA, which permeates their conduct, with the indication of follow-up in a care network with the expectation that the autonomous search will be sufficient for medicalized care.

SA care practices follow the influence of hospital-centric history^(21)^. Forwarding needs to be redefined: from irresponsible to implicated, where professionals must be responsible for addressing the request, ensuring that the indicated service will be able to accommodate children and adolescents, being part of a network that, in fact, will act as a follow-up to the ER’s initial conduct^(9)^. Furthermore, medicalization of care is seen early, with the use of drugs as a way of treating emotional aspects without reflecting that their effects are long-term, requiring adherence to therapy which, in itself, without integration into a context broad, will not be able to minimize mental distress^(13)^.

Another aspect is the view that mental health demands should be met exclusively by psychologists or psychiatrists. However, professionals from different backgrounds can be promising in mental health care, as long as they strive for a non-judgmental welcome^(4,22)^. For instance, communication is indicated, by international guidelines, as an essential competence of health professionals in all contexts^(23)^. In clinical practice, there are numerous efforts by teams to train in this skill, but always focusing on diagnoses and communicating bad news, not focusing on therapeutic communication.

In the present study, “drawing attention” was attributed as a triggering factor for SA. However, we reflect: what does attracting attention mean? Theoretically, it is known that SA is the result of intense mental distress, however, in this study, is reduced to the pathological idea of childhood and adolescence^(13)^, with the transition between “problematic” phases where any emotional expression is seen as a desperate attempt to draw an adult’s attention, or reduced to the idea that “this phase is just like that”, minimizing mental health demands, through transitional behaviors^(5,22)^. This idea permeates all subcategories and can be associated with Symbolic Interactionism, where individuals do not respond to the world as it is, but rather based on the reality defined by them^(17)^, with professionals attributing common sense beliefs to their actions, such as the view that family reprisals would be necessary to reduce attempts to “draw attention”. Furthermore, these beliefs can permeate the vision of other actors in society, such as family members.

In a study carried out with family members of children with SA, it was noted that they were unable to distinguish between children’s mental health needs and behaviors^(3)^. This may be a common reality and should not be labeled as negligence, because, just as professionals do not demonstrate knowledge about the topic, families also experience the same, as seen in NT10’s speech (Chart 1). Furthermore, the family and the support network play a crucial role in the care process in SA, both as an active participant, focusing on care actions, and as a caregiver, who will mediate children’s entry into health services on a longitudinal basis^(1,2,22)^, and the necessary tools must be offered. It is necessary to mediate collective efforts to transmute the idea of “drawing attention” to a warning sign for mental health problems that are being manifested and must be welcomed^(6)^.

Despite the publication of hospitalized children’s rights, in this study, there is a need to translate these rights into clinical practice, contemplating the right to protection, life and health without any form of discrimination, receiving all available therapeutic resources and relying on soft care technologies^(24)^. Judgment is still dominant, reflecting the historical construction of stigmatization of individuals with mental health problems^(1)^. This aspect can contribute to self-stigmatization and withdrawal from services, an aspect referred to as one of the main barriers to seeking care^(1,25)^.

One of the guiding principles of PSMIJ indicates the permanent construction of the network, which presupposes the idea that care is not limited to technical or specialized interventions, with the need for interconnection in a broad network with intersectoral services^(9)^. To this end, the reflections listed here must also be integrated into these services, aiming to avoid further separation of children and adolescents from RAPS. However, the literature shows that professionals in these services also experience limitations, as seen in a Brazilian qualitative study carried out with 12 professionals from primary care and a Psychosocial Care Center (CAPS - *Centro de Atenção Psicossocial*), where they reported that they did not feel prepared in SA care, as there was no pool of professionals with experience working. Furthermore, they mentioned impasses in the organization of the care network, with a lack of professionals, lack of communication between levels of care and discontinuity of care^(12)^, statements that corroborate this study. This aspect demonstrates that the problem of welcoming SA goes beyond care in ER.

It is expected that children and adolescents will be welcomed at CAPIJ, but these only exist in cities with more than 70 thousand inhabitants; when not present on the network, this audience is referred to CAPS that assist adults^(12)^. It is worth reflecting on how Brazilian cities that do not have this device on the network organize themselves, as well as other equipment in the territory where children are immersed, requiring further studies. Here the co-participating institution is in the central region of SP, with the availability of CAPSIJ, making articulation with matrix support, management and referral possible. It is noteworthy that both devices are part of the RAPS, with their articulation being a fundamental premise of public policies^(11)^.

Another focus should be given to the emotional reactions generated in the team by the process of attending to SA. In this study, the emotional reactions reported may be associated with the ER, which has its architecture and professional staff created to promote life^(2,3)^. Furthermore, professionals realize that “saving lives” is synonymous with good care, without reflecting subsequent aspects. A professional (SW4, Chart 2) indicates that time was an important aspect in mitigating her emotional reactions. From an interactionist perspective, it is observed that the past provides support for present actions^(17)^, but new studies are needed, since having no reaction can also be transmitted to practice with the coldness of care marked by indifference and withdrawal.

Symbolic Interactionism indicates that the meanings attributed to a phenomenon can be modified through an interpretative process used by individuals to deal with the context^(17)^. Thus, it is reflected on the change strategies indicated by professionals that, if worked on, can be predictors for change. The majority of professionals indicate institutional protocols as the key strategy, however, implementing a protocol that indicates welcoming actions, without preparing the team and maintaining constant education and practical and personal redefinitions, may not have results. Another point is the appointment of a single welcoming team, which is a restrictive measure.

Continuing education is essential, already recognized by the WHO as a pillar of the Live Life guideline^(4)^. It is reflected that it must be started during the undergraduate period^(26)^, however, in practice, it is observed that teachers tend to restrict the SA approach with students for fear of triggers. However, it is already recognized in the literature that the fact is the opposite, as talking about the topic can be an opportunity for discussion and reducing individuals’ anxiety when faced with suicidal thoughts^(27)^. Starting students’ improvement can be a predictor for qualified future care, with continuity when they become professionals, providing the transformation of experiences with a new interaction in the face of the phenomenon^(17)^. Furthermore, as reflected in the subcategory: Giving new meaning to care: institutional or personal changes?, if professionals do not identify their approach to SA as a problem and are motivated to change their vision, practice will continue to be permeated with stigma.

It is essential to reflect on strategies to improve child and adolescent mental health care in emergency services. Paths have already been taken, but the promulgation of legal texts does not alone bring about the necessary changes, with implementation research being of vital importance, seeking the power of RAPS as a comprehensive care policy linked to the territory, overcoming asylum remnants and sustaining the condition of children and adolescents as subjects of rights who deserve to be cared for and respected, regardless of condition and sector. In the current context of Brazilian health, the study of this topic is necessary and promising as the beginning of a line of investigations aimed at providing practical changes.

This study has as a limitation the data collection carried out in only one institution and with greater representation of the nursing team, which may reduce the phenomenon to the perception of a cultural group of professionals. Another limitation was the lack of analyzes that considered the differences between the profiles and characteristics of professionals in terms of understanding SA care, which could contribute new data to the proposed discussion.

## CONCLUSION

The professionals in the services studied understand the care of children and adolescents due to SA with perspectives permeated by judgment, lack of knowledge, lack of preparation and a strictly biomedical approach. A stigmatizing view of the phenomenon was perpetuated, with minimization of mental distress, with “drawing attention” being the most frequent set of words in speeches and identified as the main triggering factor for SA. They refer to emotional reactions, such as frustration, resulting from care that is seen as a specific part of a longitudinal demand. They indicate change strategies, but reflect the extent to which changes should be institutional or personal. It is hoped that this study can provide the reader with reflections on clinical practice and strategies for changing paradigms, making SA care an emerging priority.
